# Implementation of the Vapor–Liquid Equilibrium On the Kinetic Model for the Oligomerization of Olefins

**DOI:** 10.1021/acs.iecr.5c03201

**Published:** 2025-10-14

**Authors:** Tomás Cordero-Lanzac, Zuria Tabernilla, Eva Epelde, Andrés T. Aguayo, Javier Bilbao, Ainara Ateka

**Affiliations:** a Department of Chemical Engineering, 16402 University of the Basque Country UPV/EHU, PO Box 644, Bilbao 48080, Spain; b IKERBASQUE, Basque Foundation for Science, Bilbao 48080, Spain

## Abstract

The oligomerization of light C_2_–C_4_ olefins has emerged as one of the most studied processes for the sustainable production of different refinery cuts of interest as green fuels, such as gasoline, diesel, or aviation fuels. However, oligomerization is a complex process in which the presence of a gas and liquid phase (heavy oligomers) leads to unique phenomena in this reaction: an apparent initial deactivation of the catalyst due to the pore filling with retained oligomers and a steady state of catalyst constant activity. These features make it difficult to develop robust kinetic models to simulate the reactor and upscale the process. Herein, a lump kinetic model has been developed for the simulation of 1-butene oligomerization in a packed-bed reactor using a catalyst of the HZSM-5 zeolite embedded in a mesoporous γ-Al_2_O_3_ matrix. The computation methodology is based on a one-dimensional three-phase reactor model combined with vapor–liquid equilibrium calculations and a concentration-dependent deactivation equation. The compounds in the gas phase were considered reactive, while those in the liquid phase were assumed to be retained in the catalyst pores. The model suitably fitted the experimental data obtained at 40 bar, different temperatures (150–250 °C), and space-time values up to 5.7 g h mol_C_^–1^. The proposed model reconciled some of the observations in literature for oligomerization, such as the effect of the retained liquid as deactivation kinetics distorted by pore diffusion resistance or the higher stability (high remanent activity) in the presence of the liquid flow.

## Graphical abstract

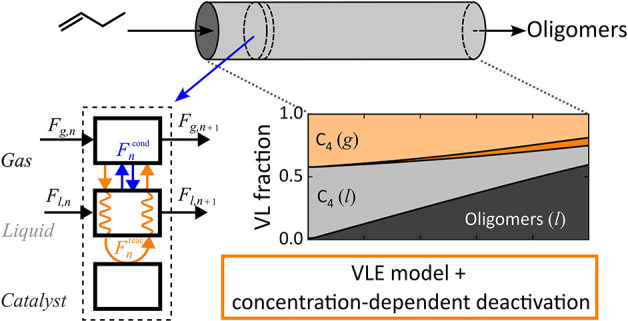

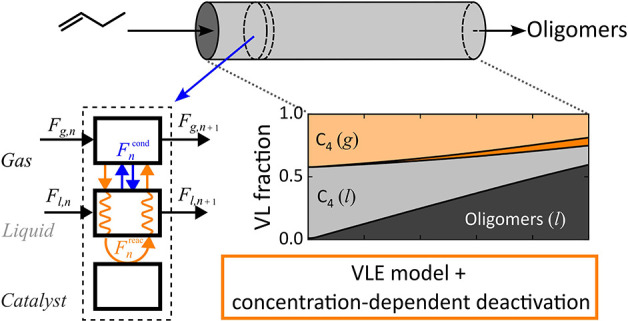

## 1 Introduction

The oligomerization of light olefins (C_2_–C_4_) into clean and sulfur-free green fuels is of significant strategic interest in the energy transition period, due to the increasing prospects of sustainable and more energy-efficient routes to produce light olefins.^1,2^ Currently, the main routes of light olefin production are steam cracking of petroleum naphtha or ethane from shale gas (with ethylene as the main product)^3,4^ and fluid catalytic cracking (FCC) (oriented toward propylene production).^5^ However, there is an increasing interest and encouraging results in processes for a greener production of light olefins among the carbon capture, utilization, and storage (CCUS) strategic areas of the chemical industry. Using captured CO_2_ or syngas from the gasification of renewable sources, light olefins are produced from methanol or dimethyl ether-mediated CO_2_/CO hydrogenation in two stages^6^ or in a single step using OX-ZEO catalysts.^7,8^ Short and long-chained olefins can also be produced via the modified Fischer–Tropsch (FT) reaction with selective catalysts.^9,10^ For the large-scale production of green fuels from these light olefin oligomers, heterogeneous catalysts offer advantages over homogeneous ones. The most used catalysts are based on zeolites such as HY, HBeta, or HZSM-5,^11,12^ sometimes doped with Ni to improve their activity.^13^ It is worth mentioning that HZSM-5 zeolite was already used in the Mobil olefins to gasoline and distillates (MOGD) process.^14^

A remarkable characteristic of oligomerization reactions over heterogeneous catalysts is their well-known diffusion constraints. Generally, the low apparent activation energy values observed are attributed to the limitations of intrazeolite diffusion due to the presence of the oligomers retained in the pores of the zeolite. This diffusional limitation increases with the oligomer size.^15–17^ Moreover, retained oligomers hinder the access of reactants to the active sites,^18^ having a direct influence on the apparent deactivation of the catalyst. Thereby, a decrease in the oligomerization rate is reported as bigger oligomers are formed and retained within the zeolite pores.^15,19,20^ This (apparent) deactivation is nonetheless reversible, and the catalyst recovers the initial activity through sweeping with inert gas.^17,21,22^ This contrasts with most acid-catalyzed reactions, which normally deactivate by coking and require a combustion to recover catalyst activity by burning the coke. In the nonsteady state, the apparent deactivation rate decreases with time and tends toward a steady state, where a pseudoequilibrium oligomerization rate is observed. The time to achieve the steady state depends on the reactant, reaction conditions, and, most importantly, the porous texture of the catalyst.^16,17,19–21^ Other experimental observations supported the relevant role of the retained oligomers, the liquidlike phase, in the oligomerization of light olefins. Among those, one can highlight the higher apparent conversion of ethylene than that of butene due to the lack of big oligomers,^23^ or the better stability of the zeolite with the presence of a liquid phase.^24,25^ This theory is also reinforced by Jan et al.,^26^ who rationalized the mass-transfer limitation of reactants and products due to the formation of a liquid film on the zeolite crystals during the oligomerization of ethylene.

The development of kinetic and reactor models for the oligomerization of olefins should therefore consider some specific phenomena occurring in parallel to the reactions. Especially, the presence of diffusional limitations (within the zeolite and catalyst particles) causes the fast initial apparent deactivation of the catalyst. This is related to the different states (vapor or liquid) of the compounds and their reactivity as vapor or liquid, which hinders the kinetic modeling using the habitual criteria for catalytic processes with reactants and products in the gas phase. Some kinetic studies of olefin oligomerization over different zeolites in the literature explain the peculiar performance of the reaction in a similar manner to heterogeneous catalytic processes with internal diffusion limitations,^15,17,27^ while others proposed deactivation kinetics to quantify the evolution of the product distribution with time in the nonsteady state.^28–30^

Satterfield and Stenger^31^ studied the effect of the presence of gas or liquid phase compounds in different reactions (isomerization and hydrogenation) over porous catalysts, observing differences in the conversion with the presence of a liquid phase. These authors found different adsorption strengths for the gas and liquid phases and hindered reaction rates for the compounds in the gas phase due to the adsorption of the liquid on the acid sites, acting as an inert site in the reaction. Based on these premises, a vapor–liquid equilibrium (VLE)-based kinetic model for the oligomerization of olefins is a reasonable approach to the experimental evidence discussed above. Likewise, it may well be hypothesized that the compounds in the gas phase would predominantly react over the active sites of the catalyst, while the formed liquid phase would be retained in the pores and weakly adsorbed, thus limiting site accessibility. The different roles of the gas and liquid phase have been successfully applied in VLE-based kinetic models in different multiphase catalytic processes at high pressure, with different reactivity of the compounds in each phase; for example, in simulations of thermal cracking reactors,^32,33^ hydrocrackers of oil fractions,^34–37^ and reactors of biomass-derived oil hydrodeoxygenation.^38^

In this work, we propose a lump kinetic model, implementing VLE calculations, for the oligomerization of 1-butene over an HZSM-5 zeolite-based catalyst with a mesoporous γ-Al_2_O_3_ matrix, using experimental results obtained in a packed-bed reactor. The consideration of the effect of the oligomerization conditions in the vapor–liquid equilibrium is the key factor to quantify the different reactivity of the components throughout the reactor and their role in the reaction according to their physical state. The consideration of VLE calculations on the deactivation kinetics is an original contribution in the oligomerization of olefins. The model approaches the role of the different phases in the catalyst and predicts the results in the nonsteady state and in the subsequent steady state (of greater interest for scale-up).

## 2 Experimental Section

### 2.1 Catalyst

The catalyst used for 1-butene oligomerization runs was prepared by agglomeration of an HZSM-5 zeolite to meet industrial specifications. The used protocol was previously described.^39^ Briefly, a previously calcined HZSM-5 zeolite with a SiO_2_/Al_2_O_3_ ratio of 30 (active phase, Zeolyst International) was mixed with a colloidal dispersion of α-Al_2_O_3_ (inert filler, Alfa Aesar) and pseudoboehmite (binder, Sasol Germany), targeting a final composition of 50/18/32 wt % of zeolite/filler/binder. The slurry was extruded and dried overnight. Afterward, the extrudates were calcined at 575 °C for 2 h using a temperature ramp of 5 °C min^–1^. During the calcination, the pseudoboehmite is converted to γ-Al_2_O_3_, providing the final catalyst with a mesoporous matrix of very weak acidity (inactive for 1-butene oligomerization). The enhanced diffusion of the oligomers through the catalyst matrix avoids pore blockage and favors a steady state of operation to be obtained during the 1-butene oligomerization.^21,30^ The main physicochemical properties of the catalyst are summarized in Table S1 of the Supporting Information.

### 2.2 Equipment and Reaction Conditions

Experimental runs were carried out in a high-pressure unit (PID Tech. & Eng.) provided with a packed-bed stainless steel reactor, heated by a ceramic oven. The catalyst particles were mixed with SiC to maintain a constant bed height and isothermal conditions in all runs. Prior to the reactions, the catalyst was pretreated at 450 °C and atmospheric pressure under a constant flow of 50 cm^3^ min^–1^ He for 3 h. Afterward, temperature and pressure were set to the reaction conditions, and 1-butene was fed using a low-temperature liquid pump to be able to operate at high pressure. The used operation conditions were: 40 bar, 150–250 °C, a space time of 0.5–5.7 g h mol_C_^–1^, and time on stream up to 20 h. Reaction products were sent to a cold trap (Peltier cell), where the liquid and gas products were separated. Gaseous products were analyzed in-line using an Agilent 3000A microGC, while liquid products were analyzed *ex situ* in a GC (Hewlett-Packard 6890 Series II) and GC × GC/MS (Agilent Technologies 7890A gas chromatograph) coupled in-line with an XL MSD mass spectrometer (Agilent 5975C Series GC/MSD). From the analysis of the gas and liquid effluents, 1-butene conversion (*X*) was defined on a carbon basis as

$$X=\frac{F^{\mathrm{in}}-F}{F^{\mathrm{in}}}100$$ 1

where *F* ^in^ and *F* are the carbon molar flow rate of 1-butene in the feed and at the outlet of the reactor, respectively. Due to the complexity of the analysis (mixture of hydrocarbons sampled in gas and liquid phase at different time intervals and analyzed in three GCs), mass balances were closed ca. 90–95 wt% in most runs, which have been indicated with the error bars.

## 3 Methods

### 3.1 Kinetic Model

The oligomerization mechanism proceeds through the protonation of an adsorbed olefin on a Bro̷nsted acid site to form a carbocation intermediate. This intermediate adds another olefin, forming a larger carbocation intermediate, whose subsequent desorption yields the olefin product. The oligomers can also undergo side reactions of double bond and skeletal isomerization, cyclization, cracking (β-scission) or hydride transfer reactions, although they are less favored at high-pressure conditions.^40^ Experimentally, we observed butane as the only paraffin and negligible aromatics and C_16+_ compounds, suggesting an insignificant contribution of side reactions and oligomerization of the tetramer (C_16_). Figure 1a shows the relative average concentration of compounds in the reactor effluent for all of the performed runs. As expected, the main products were the dimer and trimer of 1-butene oligomerization (blue in Figure 1a). A reaction network of 7 lumps with 6 reaction steps was defined (Figure 1b). Apart from the butene lump (C_4=_), all the direct olefin products from the oligomerization with butene were considered (C_8_ dimer, C_12_ trimer, and C_16_ tetramer). Despite their lower concentration, C_3_ and C_5_ hydrocarbons were observed, which are the main products of octene cracking. Likewise, two lumps of C_6_–C_9_ and C_10_-C_15_ olefins were considered from oligomerization-cracking reactions. The reaction network in Figure 1b distinguishes the main reaction routes: the direct oligomerization pathway, where butene is the main reactant forming the dimer, trimer, and tetramer in sequenced steps (reaction steps 1–3), and the formation of secondary products via cracking/oligomerization-cracking routes (reaction steps 4–6).

**Figure 1:**
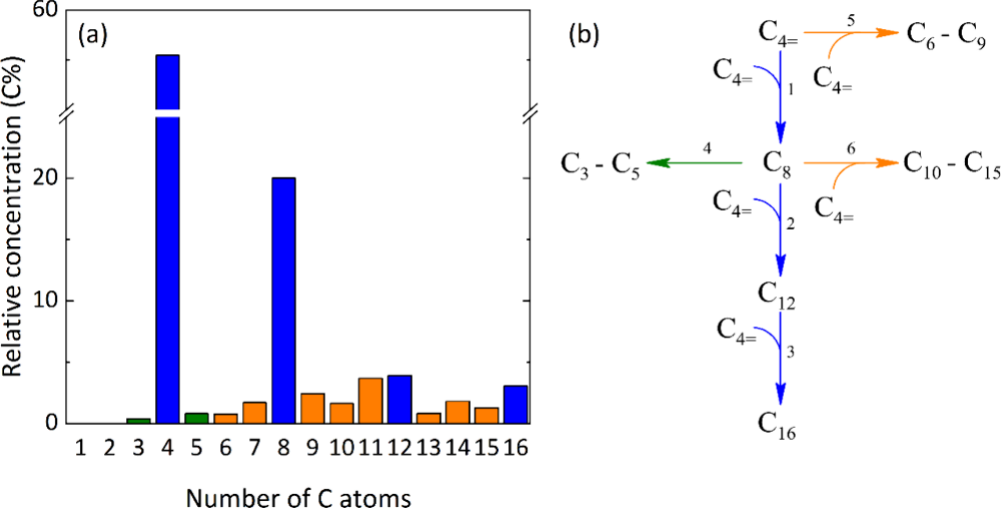
(a) Relative average concentration of products observed experimentally and (b) proposed lump-based reaction network for the oligomerization of 1-butene considering the main oligomerization (blue path) route and side reactions of cracking (green path) and oligomerization-cracking (orange paths).

The Eley–Rideal postulate was used to describe the reaction rate of each *j* step of the reaction network (*r*_*j*_), as it has been consistently done in the literature.^15,18,27,41,42^ Therefore,

$$r_{j}=\left(r_{j,0}\right)a=\frac{k_{j}\prod_{ij}f_{ij}}{1+K_{\mathrm{ads}}f_{C_{4}}}a$$ 2

where (*r*_*j,*0_) is the reaction rate of the *j* step at zero time, *a* is the catalyst activity, *k*_*j*_ is the kinetic constant, *f*_*ij*_ is the fugacity of the *i* lump involved in the *j* reaction step, *f*_C4_ is the fugacity of 1-butene, and *K*_ads_ is the adsorption equilibrium constant of 1-butene. All kinetic constants depend on the reaction temperature following the reparametrized Arrhenius equation,

$$k_{j}={k_{j}}^{*}\exp\left[-\frac{E_{j}}{R}\left(\frac{1}{T}-\frac{1}{T^{*}}\right)\right]$$ 3

where *k*_j_* is the kinetic or deactivation constant at the reference temperature T* (200 °C in this work) and *E*_*j*_ is the apparent activation energy of the *j* reaction step. Herein, only two apparent activation energy values were considered for the 6 reaction steps detailed in Figure 1b to avoid overparameterization: one for the oligomerization reactions and another for the cracking reactions.

The decrease in the conversion with time during a nonsteady period of apparent deactivation is attributed to the retention of heavy oligomers within the catalyst pores that slows down the reaction rate rather than to the formation of solid coke deposits.^17,21,22^ In eq 2, the apparent activity (*a*) is calculated as the ratio between the reaction rate of a given *j* reaction step at time *t* and the corresponding initial rate of that reaction for the same composition of the reaction medium.^43^ In this work, and due to the nature of deactivation (physical phenomenon of pore blockage by retained oligomers), only nonselective deactivation models were used, assuming that the apparent deactivation affects equally to all steps of the reaction network in Figure 1b. The few works of oligomerization kinetic modeling in literature that considered deactivation kinetics used empirical concentration-independent equations.^22,28,29^ Here, we extend the study of deactivation to different models: independent of the reaction medium concentration (deactivation equation "i" in eq 4), dependent on the gas-phase product concentration (deactivation in series with the extent of the main reaction, deactivation equation "s" in eq 5), dependent on the gas-phase reactant concentration (in parallel, deactivation equation "p" in eq 6), and dependent on the gas-phase concentration of all C-containing compounds (deactivation equation "a" in eq 7).

$$-\frac{da}{dt}=k_{d}a$$ 4

$$-\frac{da}{dt}=k_{d}\underset{p}{\sum }f_{p}a$$ 5

$$-\frac{da}{dt}=k_{d}f_{C_{4}}a$$ 6

$$-\frac{da}{dt}=k_{d}\left(f_{C_{4}}+\underset{p}{\sum }f_{p}\right)a$$ 7

where *k*_*d*_ is the deactivation constant, which also follows the Arrhenius equation (eq 3) and *f*_*p*_ is the fugacity of the C-containing products, respectively.

After the initial period of fast apparent deactivation, a (quasi)­steady conversion is experimentally observed.^12,15,20,21,28–30,44^ Consequently, a steady-state activity was considered in the kinetic models reported before.^15,30,45^ Herein, to simulate the steady-state period, activity was considered as a discontinuous function of time, independently of the deactivation equation used (eqs 4–7),

$$\frac{da}{dt}=0\;\mathrm{for}\;t>t_{ss}$$ 8

where *t*_*ss*_ is the time at which steady state was experimentally observed (*vide infra*).

### 3.2 Reactor Model

A reactor model was developed to fit the kinetic parameters with the experimental results and simulate the reactor performance by using these parameters. The high-pressure packed-bed reactor was simulated using an original one-dimensional three-phase reactor model, considering the vapor–liquid equilibrium (VLE) and the reaction of the gas-phase components on the catalyst acid sites (according to the reaction scheme in Figure 1). Figure 2 shows a schematic representation of the *N* infinitesimal volumes of the packed-bed reactor. The feed (*F* ^in^) equilibrates fast into the gas and liquid phase (*F*_*g*_ and *F*_*l*_, respectively), both of which are assumed to move with the same linear velocity. Although this is an approximation, it is evident that the liquid phase moves, and it is beyond the scope of this work to determine the exact fluid dynamics of the reactor. The component of the gas phase reacts on the surface of the catalyst, which is hindered by the presence of oligomers in the liquid phase. This restriction is considered with the deactivation equations detailed in the previous section. In each *n* infinitesimal volume, a certain molar flow of each compound is condensed to the liquid phase (*F*_*n*_^*cond*^) and a certain molar flow of each compound in the gas phase reacts over the catalyst (*F*_*n*_^*reac*^). These molar flow rates defined for clarity in Figure 2 are computed on the source terms of the mass conservation equations, as detailed below.

**Figure 2:**
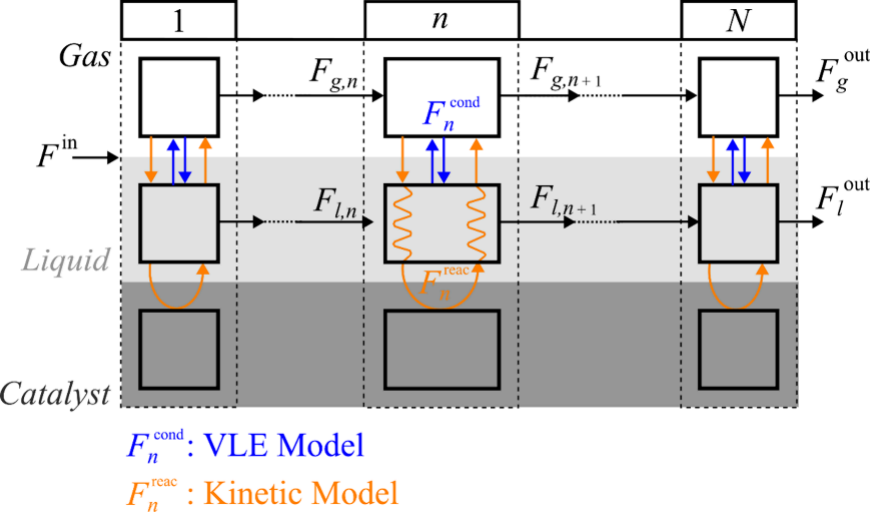
Schematic representation of the three-phase reactor model for *N* infinitesimal volumes of the packed-bed reactor.

The mass conservation equations in the gas and liquid phases were solved for each *i* lump of the reaction medium. Because of the experimental setup used, with a suitable and constant bed length throughout all the experiments where the catalyst particles were mixed with SiC particles of a suitable size, isothermal and isobaric conditions were assumed for the model. Therefore, if we assume a pure convective transport (pure plug flow) with negligible temperature variations and pressure drop, the system of partial differential equations for the evolution with time (*t*) and position (*z*) of the gas and liquid phase molar fractions (*y*_*i*_ and *x*_*i*_) is described as follows,

$$\varepsilon_{b}\frac{\partial y_{i}}{\partial t}=-\frac{\partial \left(v_{g}y_{i}\right)}{\partial z}+\frac{ZRT}{P}\rho_{b}r_{i}-\Omega_{i}$$ 9

$$\varepsilon_{b}\frac{\partial x_{i}}{\partial t}=-\frac{\partial \left(v_{l}x_{i}\right)}{\partial z}+\Omega_{i}$$ 10

defined for *t* > 0 and 0 < *z* < *L*, where *L* is the catalytic bed length, ε_*b*_ is the bed porosity, *v*_*g*_ and *v*_*l*_ are the linear velocity of the gas and liquid phase, *Z* is the compressibility factor calculated from the Soave–Redlich–Kwong (SRK) state equation to correct the deviations from ideality of gas-phase heavy oligomers at high pressure, *R* is the universal gas constant, *T* is the reaction temperature, *P* is the total pressure, ρ_*b*_ is the bed density, *r*_*i*_ is the formation rate of each *i* lump, calculated as the change of the molar fraction (in carbon basis) with space time (τ),

$$r_{i}=\frac{{dy}_{i}}{d\tau }$$ 11

and Ω_*i*_ is the vapor–liquid condensation rate of each *i* lump, calculated as,

$$\Omega_{i}=k_{y}\left(y_{i}-K_{i}x_{i}\right)=k_{x}\left(\frac{y_{i}}{K_{i}}-x_{i}\right)$$ 12

where *k*_*y*_ and *k*_*x*_ are the effective apparent vapor–liquid transport constant, including the surface area of the interphase and assumed big enough as to avoid mass transfer limitations in the vapor–liquid interphase, and *K*_*i*_ is the thermodynamic vapor–liquid phase equilibrium constant for the *i* lump, calculated as,

$$K_{i}=\frac{\gamma_{i,l}\varphi_{i,l}}{\varphi_{i,v}^{m}}$$ 13

where γ_*i,l*_ is the activity coefficient of the species *i* in the liquid phase, calculated using the regular-solution model with the Flory–Huggins correction,^46^ φ_*i,l*_ is the fugacity coefficient of the pure species *i* in the liquid phase, calculated using the Chao-Seader correlation for nonpolar mixtures,^47^ and φ_*i,v*_^*m*^ is the partial fugacity coefficient of the species *i* in the vapor phase mixture, calculated from the SRK equation of state. The thermodynamic properties of each *i* lump of products were calculated from the tabulated data of the olefin closer to the average carbon number of the lump. The evolution of the linear velocity of the gas flow was solved from the total mass balance, resulting in

$$\frac{dv_{g}}{dz}=\frac{ZRT}{P}\rho_{b}\underset{i}{\sum }r_{i}-\underset{i}{\sum }\Omega_{i}$$ 14

The following initial and boundary conditions were used for solving the system of partial differential equations,

$$y\left(0,z\right)=y\left(t,0\right)=y_{\mathrm{in}}$$ 15

x(0,z)=x(t,0)=0 16

$$\{\begin{array}{c} v_{g}\left(0,z\right)=0\\ v_{g}\left(t,0\right)=v_{\mathrm{in}} \end{array}$$ 17

where *y*_in_ is the known composition of the gas phase at the inlet of the reactor and *v*_in_ is the linear velocity computed from the experimental molar flow fed to the reactor. Please note that the inlet flow was assumed in the vapor phase, but as soon as it enters the reactor, the vapor–liquid equilibrium is quickly achieved.

### 3.3 Experimental Data Fitting

The optimization of the kinetic parameters was performed with the reactor model and deactivation equations defined above, and using the in-house vector-based optimization methodology developed before.^43^ The objective function to minimize was based on the sum of square errors (SSE) between experimental and calculated values, differentiating the initial and deactivation data,

$$\mathrm{SSE}=\sum_{i=1}^{n_{l}}\omega_{i}\left[\frac{\sum_{n=1}^{n_{e,0}}{\left(z_{i,n}^{e,0}-z_{i,n}^{0}\right)}^{2}}{n_{e,0}-1}+\frac{\sum_{n=1}^{n_{e,t}}\omega_{t}{\left(z_{i,n}^{e,t}-z_{i,n}^{t}\right)}^{2}}{n_{e,t}-1}\right]$$ 18

where *z*_*i,n*_^*e*,0^ and *z*_*i,n*_^0^ are the experimental and calculated molar fraction of the *i* lump (considering vapor and liquid phases) at the outlet of the reactor at zero time on stream, *z*_*i,n*_^*e*,*t*^ and z_*i,n*_^*t*^ are the experimental and calculated molar fraction at the outlet of the reactor at *t* time on stream, *n*_*l*_ is the number of lumps, *n*_*e,0*_ and *n*_*e,d*_ are the number of unique experimental data at zero and *t* time on stream, respectively, ω_*i*_ is the weight factor for each *i* lump, inversely proportional to its average concentration, and ω_*t*_ is the weight factor (of 0.1) applied to those experimental data point at *t* > *t*_*ss*_ to increase the significancy of the fitting during the nonsteady state.

To properly compare the SSE for each fitting and avoid the local minimum, a four-step optimization of the kinetic parameters was applied. First, the genetic algorithm implemented in MATLAB was used, with a population size of 50 and a maximum number of generations of 3 times the number of parameters to optimize (33 generations). Then, the in-house developed modified Levenberg–Marquardt was applied to individually optimize the initial and deactivation kinetic constants by independent Jacobian computation,^43^ which proved a more precise minimization of the objective function in processes with catalyst deactivation. This was followed by an overall optimization of all parameters using the built-in Levenberg–Marquardt method in MATLAB. Finally, once the tentative minimum of the optimization was reached, the genetic algorithm was used again for a test of the robustness of the fitting. Each parameter was individually tested to avoid a local minimum in the optimization, which was double checked with a sensitivity analysis with a ± 20% perturbation of the parameters.

## 4 Results and Discussion

### 4.1 Effect of the Reaction Conditions

The evolution of 1-butene conversion with time, in C units, is shown in Figure 3a,b for different values of space time and at different temperatures, respectively. Detailed product distribution can be found in Figures S1–S3. Error bars were included, indicating the analytical uncertainty and the discrepancies in C balance closure. Under all tested conditions, an initial period of fast deactivation was observed, after which steady 1-butene conversion was reached. The conversion drop is steeper at higher temperatures and values of space time. This steady state has been previously observed during ethylene,^26,48^ propene,^17^ and butene^15^ oligomerization using medium-pore zeolites or mesoporous catalysts. As reported elsewhere,^49^ the time to achieve the steady state varies between 4 and 6 h in the oligomerization of 1-butene using the mesoporous agglomerated HZSM-5 catalyst used herein. The main variable affecting this time was found to be the pressure, and this agrees with the results in Figure 3a,b. We assumed that the steady state was reached when the differences in the conversion were equal to or lower than the error closing the balances. In most cases, this occurred around 6 h on stream (indicated with a dashed vertical line in Figure 3a,b), and for a cheaper computation, 6 h was selected as the most suitable *t*_*ss*_ in eq 8. It seems clear that the level of conversion also has an effect on this time, as the conversions at low space time and temperature values stabilize slightly faster (see blue squares in Figures 3a,b). A comparison between the initial conversion values with those at steady state (more relevant from the industrial point of view) is illustrated in Figure 3c,d. 1-Butene conversion follows a linear trend with space time in both cases (Figure 3c). Similarly, the expected exponential correlation with temperature is observed at initial conditions and after the steady state was reached (Figure 3d).

**Figure 3:**
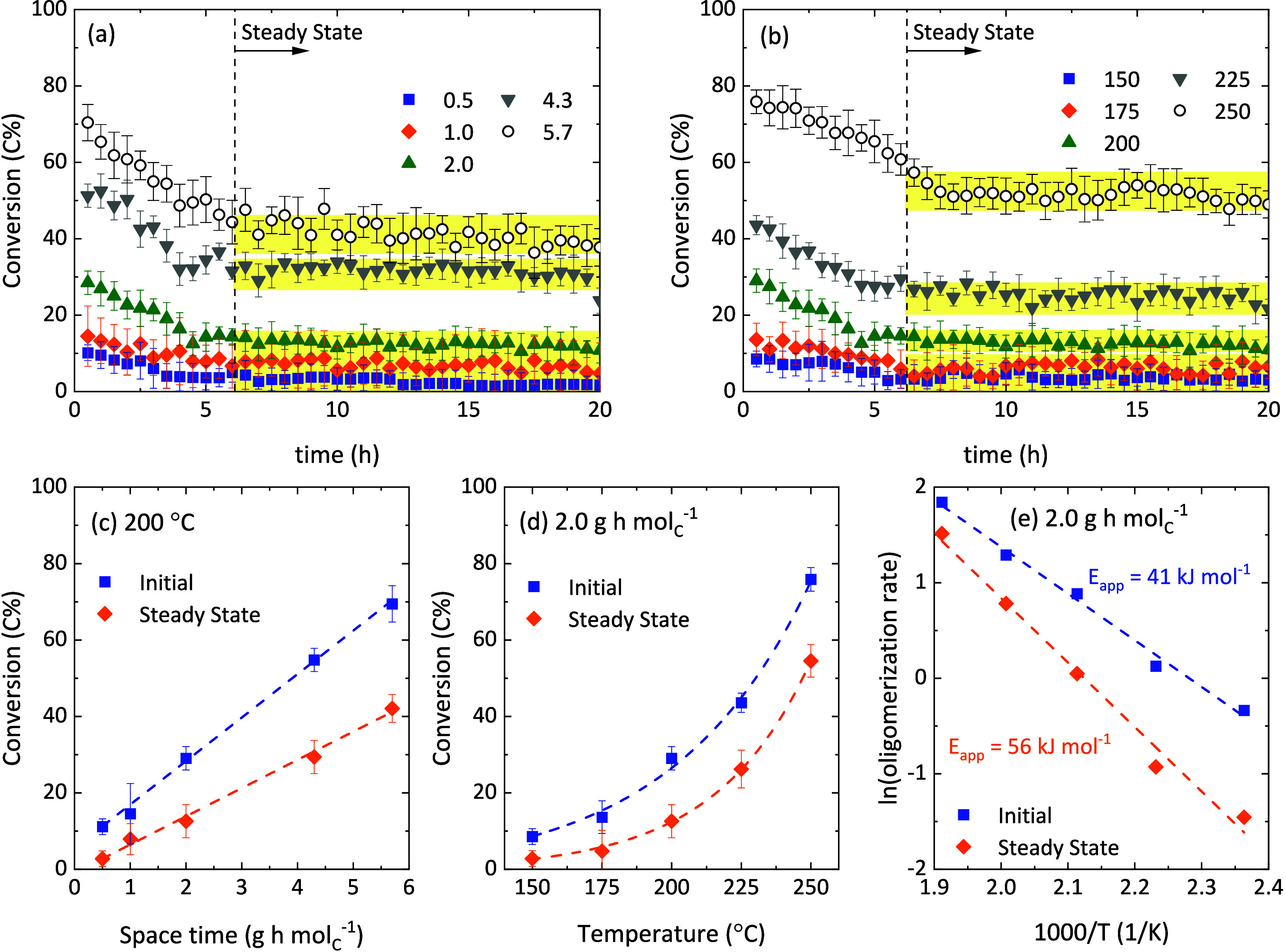
Evolution with time of 1-butene conversion (a) at 200 °C using different space-time values (in g h mol_C_^–1^) and (b) at different temperatures (in °C) using a space time value of 2.0 g h mol_C_^–1^. Evolution of the initial and steady-state 1-butene conversion with (c) space time and (d) temperature. (e) Arrhenius plot for the initial and steady-state 1-butene oligomerization using a space time value of 2.0 g h mol_C_^–1^. Total pressure of 40 bar and 1-butene partial pressure of 28 bar in all cases.

From the results at different temperatures, the oligomerization reaction rate can be calculated (considering a differential reactor), and the apparent activation energy can be estimated by means of an Arrhenius plot. Although this data treatment is an approximation, strictly correct at differential-reactor conditions where there is a linear correlation between the reaction rate and conversion, both initial and steady-state data fit the linear trends in our case (Figure 3e). The estimated values are 41 and 56 kJ mol^–1^, respectively. This approximated method is usually applied to estimate apparent activation energy values in literature, and the reported values for 1-butene oligomerization are in the range of 26–53 kJ mol^–1^. Wulfers and Lobo^15^ reported a value of 28 kJ mol^–1^ using a HBeta zeolite and conditions of low conversion. Ngandjui and Thyrion,^41^ and Nkosi et al.^50^ studied the reaction in liquid phase (high pressure) over HMordenite and Ni-HY zeolites and found apparent activation energy values of 53 and 26–33 kJ mol^–1^, respectively.

The estimation of low apparent activation energy values is also observed for the oligomerization of other light olefins, likewise assigned to mass transfer limitations (due to retained oligomers within the catalyst pores) and the presence of secondary reactions (deviating from differential-reactor conditions). Peratello et al.^18^ reported a value of 18 kJ mol^–1^ for the oligomerization of propene in the liquid phase using a SiO_2_–Al_2_O_3_ catalyst. Brogaard and Olsbye^51^ estimated, by means of density functional theory (DFT) calculations, a value of activation energy of 77 kJ mol^–1^ for the oligomerization of ethylene at 1 bar (mainly gas-phase components), while later observed experimental values of 33 and 37 kJ mol^–1^ at 4 and 26 bar, respectively, using a Ni-SSZ-24 catalyst.^52^ Similarly, Seufitelli et al.^53^ found an apparent activation energy for the consumption of ethylene over a Ni-HBeta zeolite of 44 kJ mol^–1^, while estimating values of 78 and 60 kJ mol^–1^ for the formation of hexene and octene byproducts, respectively.

### 4.2 Kinetic Model: Deactivation Kinetic Equations

The apparent initial deactivation of the catalyst in the oligomerization of olefins is obvious from the results in Figure 3 and has been quantified using kinetic equations independent of the concentration of the reaction medium.^22,28,29^ However, these “independent” deactivation equations cannot explain some of the experimental observations in the literature: the increase of the deactivation rate with the partial pressure of the reactant,^17^ the space time,^54^ and the concentration of compounds in the liquid phase.^22^ Herein, a comparison between this “independent” deactivation equation (“i”) with others dependent on the reaction medium composition is made. For that, and under our previous observation on the relevant role of pressure for the initial apparent deactivation,^21^ the composition of the gas phase was considered. Three additional deactivation equations were used to fit the experimental data: dependent on the gas-phase product concentration (in series model “s”), dependent on the gas-phase reactant concentration (in parallel model “p”), and dependent on the gas-phase concentration of all C-containing compounds (model “a”). The mathematical expressions for each model are detailed in eqs 4–7.

Figure 4a–d show the experimental data (symbols) fitting of evolution with time of the concentration of 1-butene and octene (dimer) predicted with the different deactivation kinetic equations (lines). The results correspond to different space-time values (Figure 4a,b) and at different temperatures (Figure 4c,d). The comparison of the predicted and experimental data for the rest of the compounds is shown in Figures S4 and S5. Among the evaluated models, the deactivation equation “s” (eq 5) clearly fits worse the experimental data (red and cyan lines), especially at those conditions of high conversion levels (high temperature or space-time values). This is easily explained with the simulations of the product distribution along the reactor (Figure 4e–j). Both initially (Figures 4e,g,i) and at the steady state (Figures 4f,h,j), the gas phase is mainly formed by 1-butene reactant according to the vapor–liquid equilibrium. Even at 250 °C, with the highest conversion and K-values, the reaction products (oligomers) are mainly in the liquid phase (Figure 4e,f). As deactivation kinetics is expressed as a function of the gas-phase composition, the deactivation equation “s” offered the poorest fitting of the experimental data. Because of the same reason, deactivation equations “p” and “a” (eq 6 and 7, respectively) predict equally well the evolution of the product distribution with time. Interestingly, the independent deactivation equation “i” fits the experimental data quite like these two concentration-dependent models. Most of the works in the literature used concentration-independent deactivation equations, similar to our equation “i” (eq 4).^22,28,29^ This may be a good option purely regarding the experimental data fitting. However, these integrable deactivation equations do not properly predict the known deactivation of the catalyst during oligomerization as discussed in Section 4.5.

**Figure 4:**
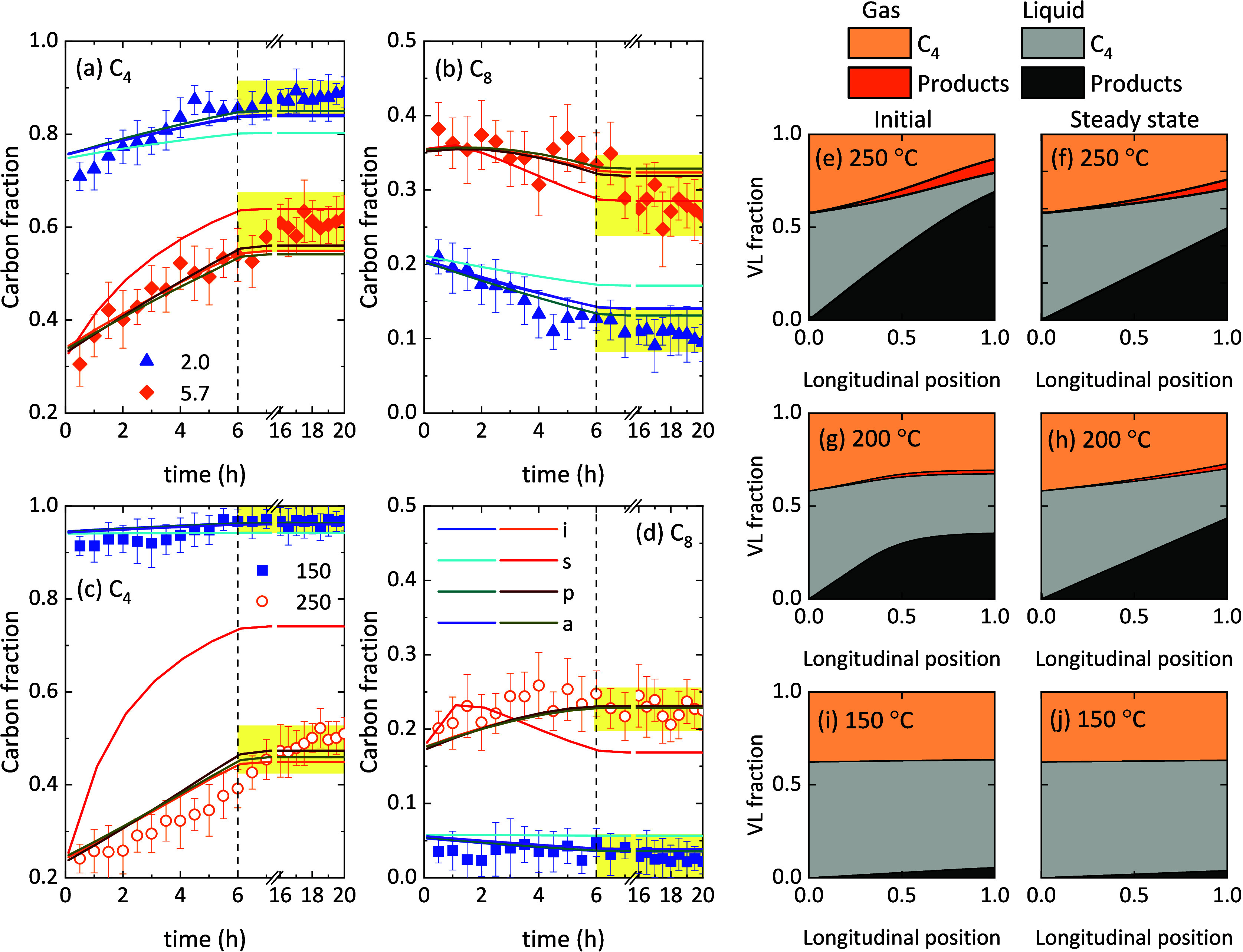
Comparison of experimental and predicted evolution with time of the (a, c) 1-butene and (b, d) C_8_ olefin carbon fractions at the outlet of the reactor with the VLE-based models and different deactivation equations (a, b) at 200 °C using different space-time values (in g h mol_C_^–1^) and (c, d) at different temperatures (in °C) using a space time value of 2.0 g h mol_C_^–1^. Simulations of the vapor and liquid fraction evolution with the reactor bed length at (e, f) 250 °C using a space time value of 2.0 g h mol_C_^–1^, (e, f) 200 °C using a space time value of 5.7 g h mol_C_^–1^ and (i, j) 150 °C using a space time value of 2.0 g h mol_C_^–1^. Total pressure of 40 bar and 1-butene partial pressure of 28 bar were found in all cases.

The kinetic parameters computed using the VLE-based model and the deactivation equation "a" are shown in Table 1. The fitting of the experimental data for all lumps is detailed in Figures S6–S8. The deviation of the parameters is also listed in Table 1, and the sensitivity analysis of every parameter is depicted in Figure S9. The most significant parameters of the model were the kinetic constant of 1-butene oligomerization to octene (first step of the reaction, *k*_1_^*^), the constant of 1-butene adsorption (*K*_ads_), and the apparent activation energy of oligomerization reactions (*E*_*O*_). The latter has a value of 42 kJ mol^–1^, like the value achieved by the simplified Arrhenius plot method of experimental data (Figure 3e) and in the range of most of the reported values in literature.^15,41,50^ From these results, it is concluded that the kinetic parameters calculated by considering the VLE better represent the kinetics of the reaction, considering the different reactivities of the compounds in the gas or liquid phase. The application of the VLE calculations also encountered a limitation of the oligomerization rate due to the pore blockage caused by the liquid, affecting the decrease in the apparent activation energy values. In their single-event microkinetic model, Toch et al. computed values of 76 and 74 kJ mol^–1^ for the activation energy of the oligomerization of ethene over Ni-SiO_2_/Al_2_O_3_ and Ni–H–Beta, respectively.^55,56^ They also estimated values of about 122 kJ mol^–1^ for the β-scission reactions during the oligomerization over the Bronsted acid sites. The oligomerization energy values cannot be directly compared due to the different reactants and sites. However, our values are almost half, suggesting conditions of internal diffusion limitation. On the other hand, the value estimated by our kinetic model for the cracking reaction (110 kJ mol^–1^, Table 1) is closer to those reported by these authors, suggesting a lower influence of internal mass transfer limitations on the cracking reaction.

**Table 1:** Kinetic Parameters for the VLE-Based Model with Deactivation Equation “a” (eq 7)

	value
*k*_1_* (mol_C_ g^–1^ h^–1^ bar^–2^)	8.6 10^–4^ ± 6.0 10^–6^
*k*_2_* (mol_C_ g^–1^ h^–1^ bar^–2^)	4.6 10^–3^ ± 6.6 10^–5^
*k*_3_* (mol_C_ g^–1^ h^–1^ bar^–2^)	1.2 10^–1^ ± 7.3 10^–3^
*k*_4_* (mol_C_ g^–1^ h^–1^ bar^–1^)	2.3 10^–3^ ± 1.0 10^–4^
*k*_5_* (mol_C_ g^–1^ h^–1^ bar^–2^)	7.7 10^–5^ ± 2.3 10^–6^
*k*_6_* (mol_C_ g^–1^ h^–1^ bar^–2^)	5.3 10^–3^ ± 8.1 10^–5^
*E*_O_ (kJ mol^–1^)	4.2 10^1^ ± 4.3 10^0^
*E*_C_ (kJ mol^–1^)	1.1 10^2^ ± 9.5 10^0^
*K*_ads_ (bar^–1^)	4.6 10^–1^ ± 3.5 10^–3^
*k*_d_* (h^–1^ bar^–1^)	8.0 10^–3^ ± 1.7 10^–4^
*E*_*d*_ (kJ mol^–1^)	1.0 10^–1^

The negligible value estimated for the apparent activation energy of deactivation (*E*_*d*_) is the other most interesting result from the model. In fact, it went to the established down limit during the optimization regardless of the deactivation model used, which was set to 1.0 10^–1^. This corroborates mathematically that the decrease in the catalyst activity is not a chemical process whose rate follows an exponential trend with temperature. Instead, the lack of correlation between temperature and deactivation rate indicates that the main cause of deactivation is the above-discussed physical phenomenon of pore blockage by retained liquid oligomers. This agrees with most of the available literature on oligomerization,^15–17^ but also with the little amount of coke and its nature observed during the 1-butene oligomerization reactions and the reactivation of the catalyst by sweeping the retained oligomers within the catalyst pores.^21^ Due to the origin of deactivation, an increase in temperature is expected to favor the formation of heavy oligomers (before their cracking at higher temperatures) but also the volatility of the compounds (increase in the K-values) and diffusivity through the zeolite channels. This opposing effect on deactivation also helps to explain the low values of apparent activation energy for the deactivation kinetics.

### 4.3 Significancy of the VLE-Based Models

From the simulation of Figure 4e–4j, one can identify the necessity of considering the VLE when oligomerization is carried out at high pressure. A fraction of each component is in the liquid phase, and this fraction is higher with increasing pressure, oligomer molecular weight, and longitudinal position in the reactor. Consequently, the simplification that the reactivity of the compounds does not depend on their gas or liquid state may represent the reality of oligomerization only at atmospheric pressure.^30^ At low pressure, the compounds (reactant and light oligomers) would be mostly in the gas phase in the reaction temperature range studied (Figure S10). Please note that the pressure and space time ranges of this simulation are out of the experimental data set, so these results are only predictions of the VLE with no reliable conversion estimations.

For the sake of comparison, we also developed and fitted the experimental data to a gas-phase kinetic model (without VLE calculations), similar to most of the data that can be found in the literature. Figure 5a,b shows the fitting of experimental data for 1-butene and C_12_ with the VLE and the gas-phase (G) models using the deactivation equations “a” and “s” in both cases. The in-series deactivation equation “s” fits better the experimental data when the gas-phase model is used because all products are wrongly assumed to be in the gas phase. Similarly, the deactivation equation “a” fits the experimental data equally well by assuming or not assuming the VLE. However, the assumption of a purely gas-phase effluent has been proven to be wrong, especially under conditions of high pressure and low temperature, when the yields of oligomers are high. The SSE (sum of square errors) for every fitting, after the four-step optimization, is detailed in Figure 5c. In all cases, the fittings of the VLE-based models are only slightly better than those achieved with the gas-phase models, except for the aforementioned deactivation equation “s”. This suggests that, mathematically, a gas-phase kinetic model may be able to fit the experimental data almost as well as considering the VLE. However, this does not mean that the model has physical meaning, especially at high pressure. The comparison of the kinetic parameters for the VLE and gas-phase models (Table S2) using the deactivation equation “a” suggests certain conclusions. Three can be highlighted: (i) the higher adsorption equilibrium constant for 1-butene (*K*_ads_) due to the absence of liquid that physically hinders the reaction, (ii) the similar values of the apparent activation energy (*E*_*O*_ and *E*_*C*_), suggesting the previously discussed internal diffusion limitation and (iii); the negligible value of the activation energy for the deactivation (*E*_*d*_), again corroborating the physical nature of the activity loss.

**Figure 5:**
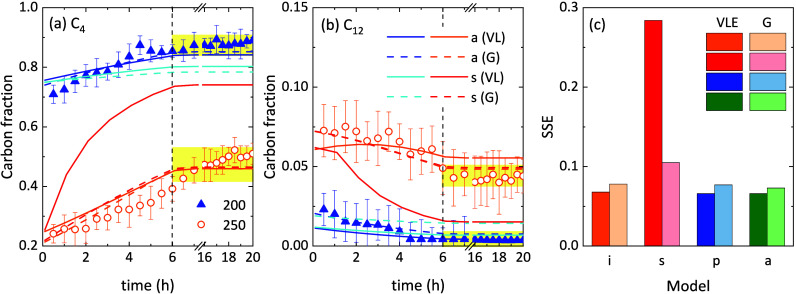
Comparison of experimental and predicted evolution with time of the (a) 1-butene and (b) C_12_ olefin carbon fractions at the outlet of the reactor and (c) sum of square errors (SSE) achieved for the best fitting of each model using different VLE and gas-phase (G) models using different deactivation equations.

### 4.4 Evolution of the Activity Profiles

The activity parameter was defined to describe the apparent deactivation of the catalyst during the reaction and is key in the simulations to ensure the quality of the fitting. Figure 6a shows the effect of the reaction conditions (temperatures and two different space-time values or reactor positions) on the evolution with time of this activity parameter, simulated by using the VLE-based model and the deactivation equation “a” (eq 7). As defined before, all curves correspond to discontinuous functions of time at 6 h, as defined in eq 8. The rate of deactivation and the steady-state activity values vary with the conditions. At the entrance of the reactor (space time 0 g h mol_C_^–1^, continuous lines), slightly faster deactivation rates are observed at the higher temperatures. However, this trend does not hold with the reactor longitudinal position. In fact, the difference between the steady-state activity at the different longitudinal positions (differences marked in Figure 6a) is a function of the 1-butene conversion and therefore the concentration of heavy oligomers. The model thus predicts that the presence of these oligomers stabilizes the catalyst, having more “available sites”, for us higher steady state activity. This agrees with the observations reported in the literature of the catalyst's slower deactivation rate due to the presence of intrapore liquid.^22,29^ Although mainly reported for ethylene oligomerization, one should expect a similar tendency, or even more noticeable, when using longer olefins. The analogous analysis was made for the VLE-based model using the deactivation equation “i”, whose fitting was very similar to the “a” model (Figure S11). Following the equation independent of reaction medium composition (eq 4), there is no effect of any reaction variable on the steady-state activity, showing less consistency of concentration-independent deactivation equations to explain the chemistry/physics of deactivation.

**Figure 6:**
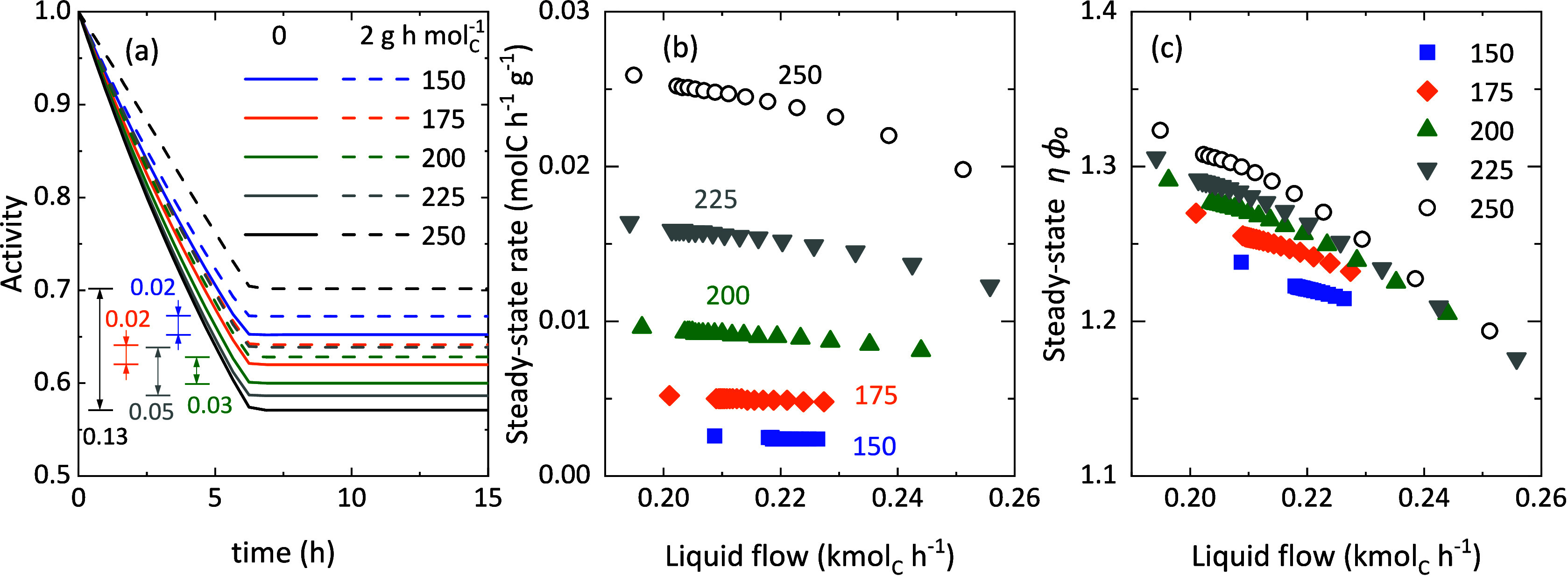
(a) Evolution with time of the activity at different temperatures (in °C) and space times values of 0 and 2 g h mol_C_^–1^. Effect of the flow of liquid products in the reactor on the steady-state (b) 1-butene self-oligomerization rate (*r*_1_) and (c) ηϕ_0_ values at different temperatures (in °C). Simulations with the VLE-based model and deactivation equation “a” (eq 7), total pressure of 40 bar and 1-butene partial pressure of 28 bar in all cases.

### 4.5 Role of the Liquid Flow

A more detailed evaluation of the peculiar performance of the oligomerization process can be carried out, focusing on the role of the presence of a liquid phase. The most significant reaction rate (1-butene oligomerization, see significance in Figure S9) shows a decreasing trend with the amount of liquid in the reactor. And before, we have already discussed the relevance of oligomer retention on the diffusion limitation of this reaction rate (see apparent activation energy values in Figure 3e and Table 1). Figure 6b also suggests that the higher the 1-butene conversion (at higher temperature), the faster the drop in the oligomerization rate. This tendency is independent of the used deactivation model in the simulation (Figures S11b and S12). Therefore, this reaction is a paradigmatic example of what Levenspiel defined as reaction kinetics with deactivation distorted by pore diffusion resistance. Following his theoretical treatment,^57^ the rate of each *j* step of the reaction network at zero time, (*r*_*j,*0_ in eq 2), corresponds to a diffusion-limited rate; thus, the rate of each *j* step at *t* time is,

$$r_{j}=\left(r_{j,0}^{\prime }\right)\eta a$$ 19

where η is the effectiveness factor due to internal pore diffusion and *r’*_*j,*0_ is the intrinsic reaction rate of each *j* step at zero time.

For a reaction affected by deactivation, the effectiveness factor depends on the evolution of the Thiele modulus (ϕ) with time. In the range of strong diffusional limitation, the effectiveness factor for a partially deactivated catalyst is inversely proportional to the product of the Thiele modulus for the fresh catalyst (ϕ_0_, before oligomers are retained) and to the square root of activity:^57^

$$\eta =\frac{1}{\phi }=\frac{1}{\phi_{0}a^{1/2}}$$ 20

According to Levenspiel's theory, this mathematical treatment led to what could be understood as a paradoxical conclusion: a decrease in the activity of the catalyst increases the effectiveness factor. However, for deactivation kinetics of order 1 (eqs 4–7) or higher, the decrease in the activity with time is faster than the increase in the effectiveness factor, thereby leading to a decrease in the apparent reaction rate with time. Figure 6c shows the evolution with liquid flow of the modified effectiveness factor (ηϕ_0_). The modified effectiveness factor decreases with the liquid flow once the steady state is reached. And by extension, it decreases following the expected trend of higher diffusion restriction with the amount of oligomers in the reaction medium. This decrease is less noticeable at lower temperatures due to the slower oligomerization rate and thus, to the lesser diffusional limitation. This interesting prediction of the oligomerization reality by the VLE-based model using the concentration-dependent deactivation equation “a” is not predicted by the concentration-independent deactivation equation “i”. Here, it is noticeable that a concentration-independent deactivation equation can fit the experimental data with a barely constant value of the effectiveness factor (Figure S11c). However, the conclusions extracted from the simulations do not seem to match the experimental observations. Consequently, the critical assessment of the model suggests that a good fitting of experimental data does not always imply a good approach to the physical reality of the catalytic processes.

## Conclusions

Vapor–liquid equilibrium (VLE) calculations were combined with the mass conservation equations in the gas and liquid phases to simulate a high-pressure packed-bed reactor for the oligomerization of olefins. The model distinguishes the different roles of the components in the gas and liquid phases in the reaction and deactivation of the catalyst. The compounds in the gas phase are reactive in the reaction mechanism, while those in the liquid phase flow through the catalytic bed without reacting. This simulates the role of the oligomers that block the catalyst pores, hindering the extent of the reaction mechanism. The integration in the model of a concentration-dependent deactivation kinetic equation allows the simulation of the nonsteady state and the steady state of the reaction, where a remanent activity of the catalyst was achieved. The evolution of the liquid flow predicted by the model reconciled some key findings in the literature: an increase in the liquid flow in the reactor increases the apparent deactivation in the nonsteady state, while the remanent activity of the catalyst in the steady state is higher due to the reported “stabilization” effect.

The proposed model was validated with experimental data for the oligomerization of 1-butene in a packed-bed reactor using a catalyst of HZSM-5 zeolite embedded in a mesoporous γ-Al_2_O_3_ matrix at low temperatures (150–250 °C) and high pressures (40 bar). The estimated kinetic parameters with the VLE-based model and the concentration-dependent deactivation equation suitably fitted the experimental product distribution during the nonsteady and the steady state (after ca. 6 h on stream), with higher significance and physical meaning than analogous gas-phase kinetic models. The low apparent activation energy for oligomerization (42 kJ mol^–1^) suggests the diffusional limitations of the reactor, which are predicted to be a function of the liquid flow with a modified effectiveness factor. Moreover, the negligible value of the activation energy for the deactivation agrees with the physical nature of the phenomenon, the partial blockage of the catalyst pores by the retained (liquid) oligomers.

The computation methodology and model proposed here can be easily applied to the oligomerization processes of different olefins and using different catalysts. Moreover, other highly studied catalytic processes, where deactivation and the presence of a liquid phase have crucial roles, can benefit from this modeling tool; for example, the Fischer–Tropsch process to convert syngas or CO_2_ into chemicals or fuels.

## Supplementary Material



## Notes

Physicochemical properties of the catalyst; additional experimental raw data; additional experimental data fitting and comparison of the models; sensitivity analysis; simulation at low pressure; kinetic parameters for the gas-phase model; and additional simulations of the VLE-based model with deactivation equations “a” and “i” (PDF)

## 1

Universidad de Malaga, Andalucía Tech., Department of Chemical Engineering, Campus de Teatinos s/n, Malaga, 29010, Spain (T.C.-L.)

## Nomenclature

a: activity parameter
*E*_*j*_: apparent activation energy of the *j* reaction step, J mol^–1^
*F*_*g*_ *, F*_*l*_: gas and liquid molar flow in the reactor, respectively, mol h^–1^
*f*_*ij*_, *f*_C4_, *f*_*p*_: fugacity of the *i* lump involved in the *j* reaction step, of 1-butene and of the C-containing products, respectively, bar
*K*_ads_: adsorption equilibrium constant, bar^–1^
*K*_*i*_: thermodynamic vapor–liquid phase equilibrium constant for the *i* lump
*k*_*j*_, *k*_*j*_*: kinetic constant of the *j* reaction step at a given temperature *T* and at the reference temperature *T**, respectively
*k*_*d*_ *, k*_*d*_ ***: deactivation constant at a given temperature *T* and at the reference temperature *T**, respectively
*k*_*y*_, *k*_*x*_: effective apparent vapor–liquid transport constant referred to the gas and liquid phase, respectively
n: elements of discretization of the packed-bed reactor
*n*_*e,0*_, *n*_*e,d*_: number of unique experimental data at zero and *t*, time on stream, respectively
*n*_*l*_: number of lumps
P: pressure, bar
R: universal gas constant, J mol^–1^ K^–1^
*r*_*i*_: formation rate of each *i* lump
*r*_*j*_, *r*_*j*,0_: reaction rate of the *j* step at *t* time and at zero time, respectively
*t, t*_*ss*_: time and time at which the steady state is reached, h
*T*, *T**: temperature and reference temperature, respectively, K
*v*_*g*_ *, v*_*l*_: linear velocity of the gas and liquid phase, respectively, m h^–1^
X: conversion
*x*_*i*_ *, y*_*i*_: molar fraction of the *i* lump in the liquid phase and gas phase, respectively
Z: compressibility factor
z: longitudinal position of the reactor
*z*_*i,n*_^*e*,0^, *z*_*i,n*_^0^: experimental and calculated molar fraction at the outlet of the reactor at zero time on stream, respectively
*z*_*i,n*_^*e*,*t*^, *z*_*i,n*_^*t*^: experimental and calculated molar fraction at the outlet of the reactor at *t* time on stream, respectively
**Greek characters**
ε_*b*_: bed porosity
ϕ, ϕ_0_: Thiele modulus at *t* time and at zero time on stream, respectively
φ_*i,l*_: fugacity coefficient of the pure species *i* in the liquid phase
φ_*i,v*_^*m*^: partial fugacity coefficient of the species *i* in the vapor phase mixture
γ_*i,l*_: activity coefficient of the species *i* in the liquid phase
η: effectiveness factor
ρ_*b*_: bed density, kg m^–3^
Ω_i_: vapor–liquid condensation rate of each *i* lump
ω_*i*_: weight factor for each *i* lump
τ: space time, g h mol_C_^–1^
