# Supplementary material for: Implementation of the Vapor–Liquid Equilibrium On the Kinetic Model for the Oligomerization of Olefins
Source: Ind Eng Chem Res. 2025 Oct 14;64(42):20207–18. doi: 10.1021/acs.iecr.5c03201 (PMC12550813; doi:10.1021/acs.iecr.5c03201)
Supplement: Supplementary file 1 [file ie5c03201_si_001.pdf]

Supporting Information

Implementation of the Vapor-Liquid equilibrium (VLE) on the kinetic model for the oligomerization of olefins

Tomás Cordero-Lanzac [a,b,*,1], Zuria Tabernilla [a], Eva Epelde [a], Andrés T. Aguayo [a], Javier Bilbao [a], Ainara Ateka [a]

[a] *Department of Chemical Engineering, University of the Basque Country UPV/EHU, PO Box 644, 48080, Bilbao, Spain*

[b] *IKERBASQUE, Basque Foundation for Science, Bilbao, Spain*

*Corresponding author: tclanzac@uma.es

[1] *Present address: Universidad de Malaga, Andalucía Tech., Department of Chemical Engineering, Campus de Teatinos s/n, Malaga, 29010, Spain*

Table S1. Physicochemical properties of the catalyst

	**Value**
N_2 adsorption-desorption	
S_{BET} (m^2 g^{-1})	266
V_{microp} (cm^3 g^{-1})	0.05
V_{mesop} (cm^3 g^{-1})	0.38
NH_3 temperature-programmed desorption	
Density of acid sites (mmol g^{-1})	0.38
Average acid strength (kJ mol^{-1})	110

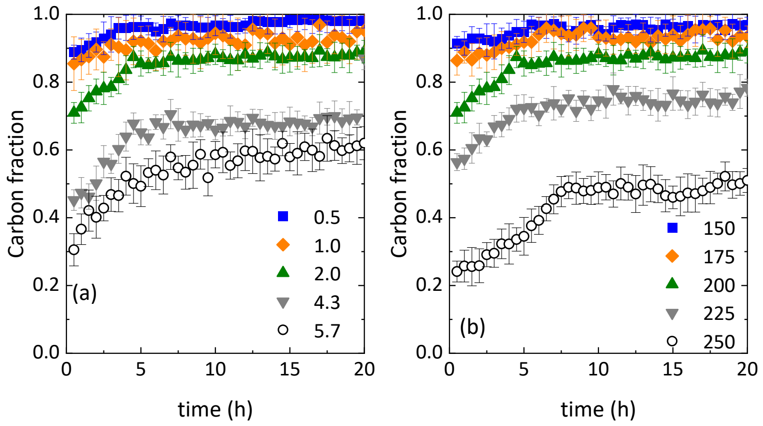

Figure S1. Evolution with time of 1-butene carbon fraction at the outlet of the reactor (a) at 200 ºC using different space time values (in g h mol_C^{-1}) and (b) at different temperatures (in ºC) using a space time value of 2.0 g h mol_C^{-1}. Total pressure of 40 bar and 1-butene partial pressure of 28 bar in all cases.

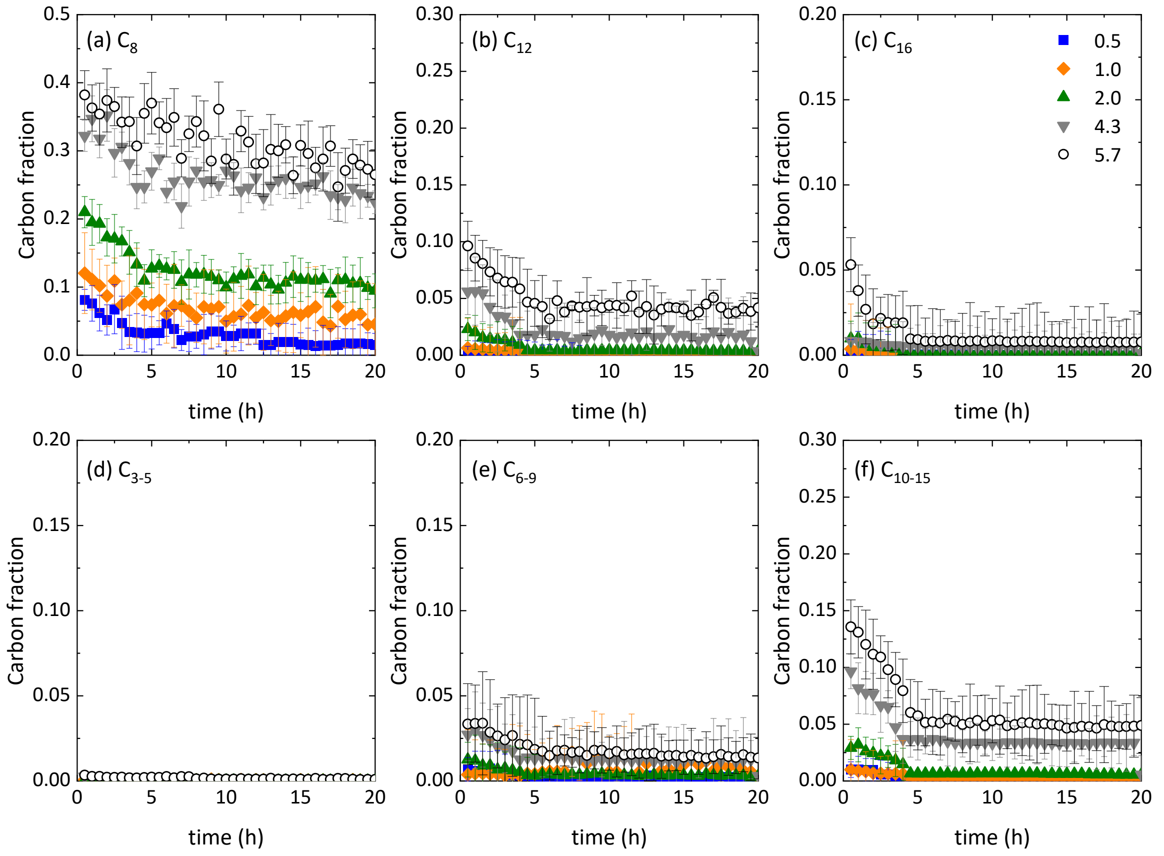

Figure S2. Evolution with time of (a) C_8, (b) C_{12}, (c) C_{16}, (d) $C_{3\text{-}5}$, (e) $C_{6\text{-}9}$, (f) $C_{10\text{-}15}$ olefin carbon fractions at the outlet of the reactor at 200 ºC using different space time values (in g h mol_C^{-1}). Total pressure of 40 bar and 1-butene partial pressure of 28 bar in all cases.

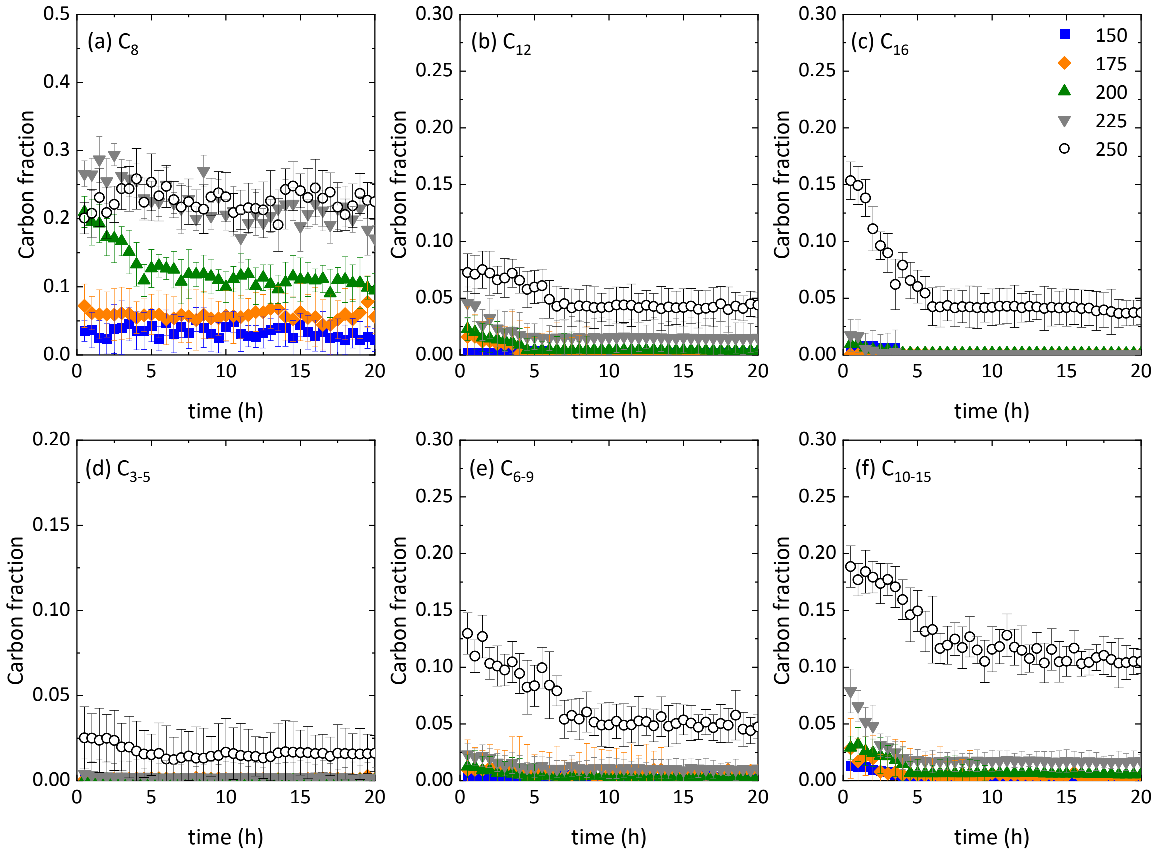

Figure S3. Evolution with time of (a) C_8, (b) C_{12}, (c) C_{16}, (d) C_{3-5}, (e) C_{6-9}, (f) C_{10-15} olefin carbon fractions at the outlet of the reactor at different temperatures (in ºC) using a space time value of 2.0 g h mol_C^{-1}. Total pressure of 40 bar and 1-butene partial pressure of 28 bar in all cases.

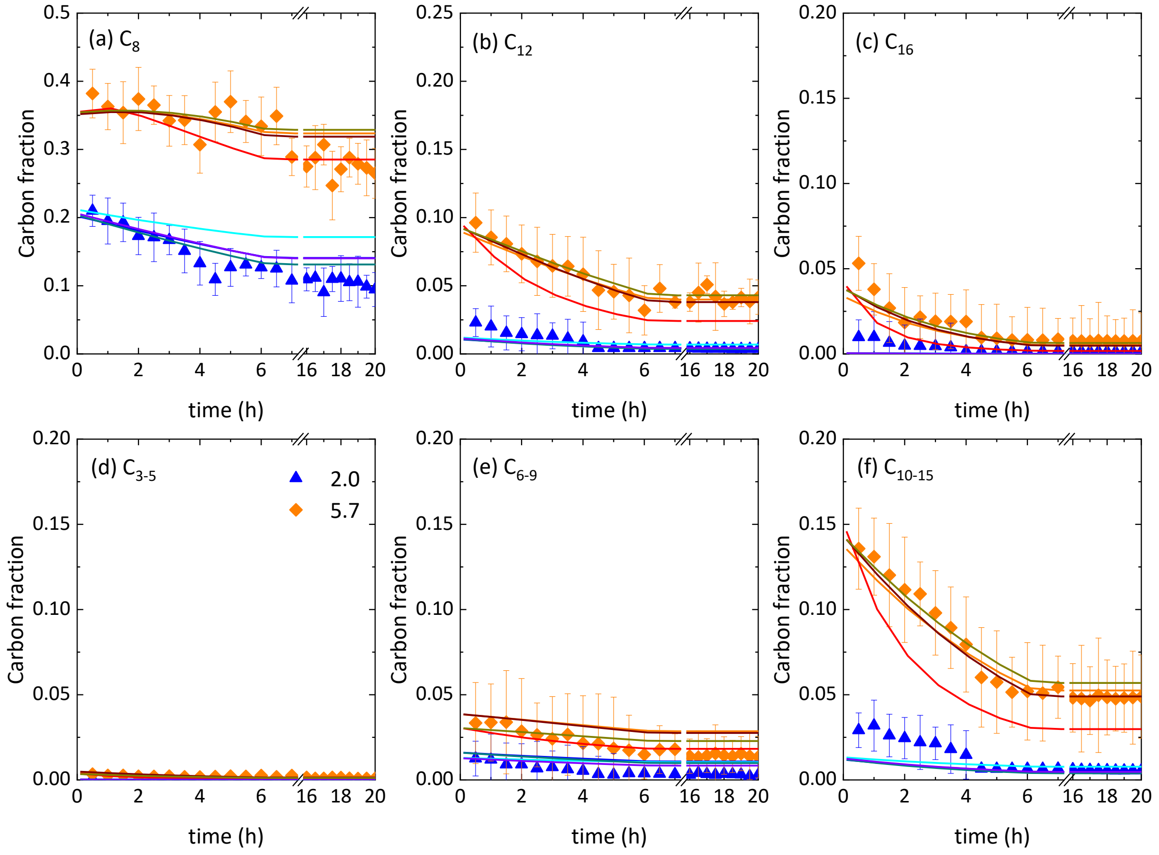

Figure S4. Comparison of experimental and predicted evolution with time of (a) C_8, (b) C_{12}, (c) C_{16}, (d) C_{3-5}, (e) C_{6-9}, (f) C_{10-15} olefin carbon fractions at the outlet of the reactor with the different VLE models at 200 ºC using different space time values (in g h mol_C^{-1}). Total pressure of 40 bar and 1-butene partial pressure of 28 bar in all cases.

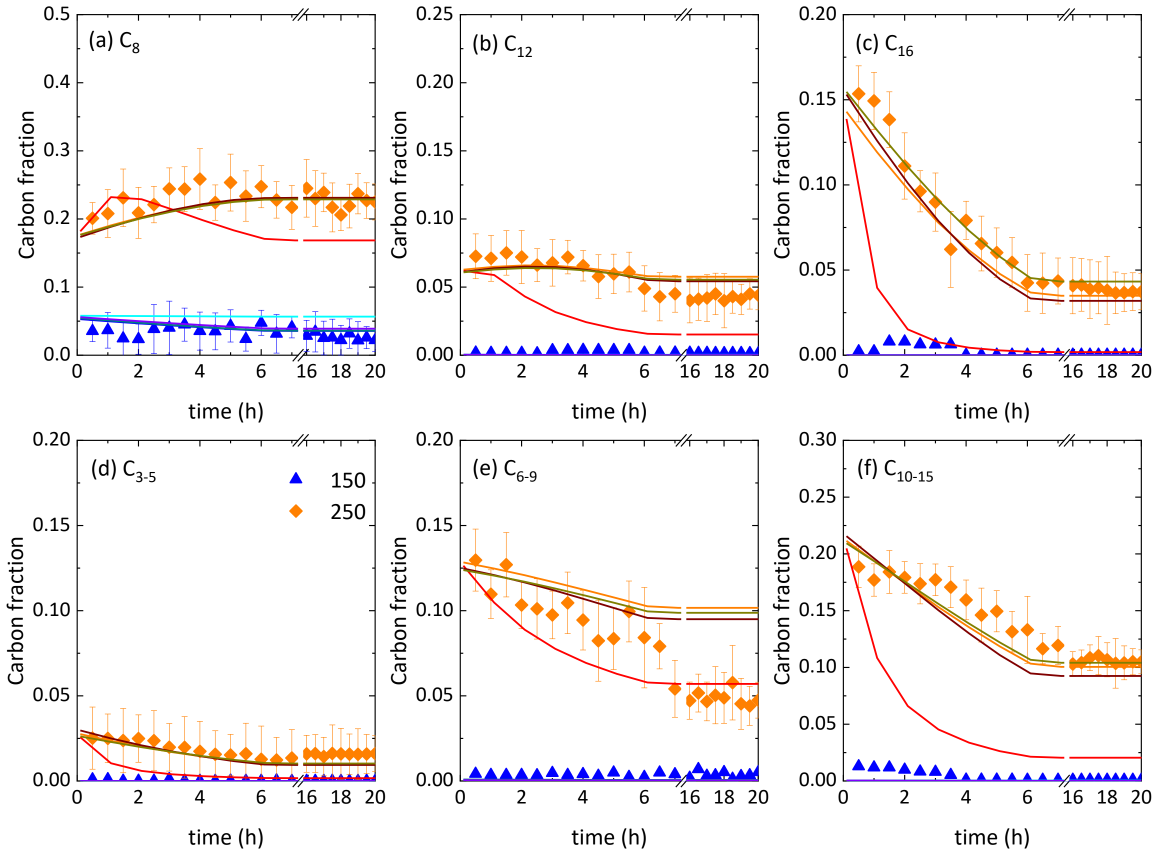

Fig S5. Comparison of experimental and predicted evolution with time of (a) C_8, (b) C_{12}, (c) C_{16}, (d) C_{3-5}, (e) C_{6-9}, (f) C_{10-15} olefin carbon fractions at the outlet of the reactor with the different VLE models at different temperatures (in ºC) using a space time value of 2.0 g h mol_C^{-1}. Total pressure of 40 bar and 1-butene partial pressure of 28 bar in all cases.

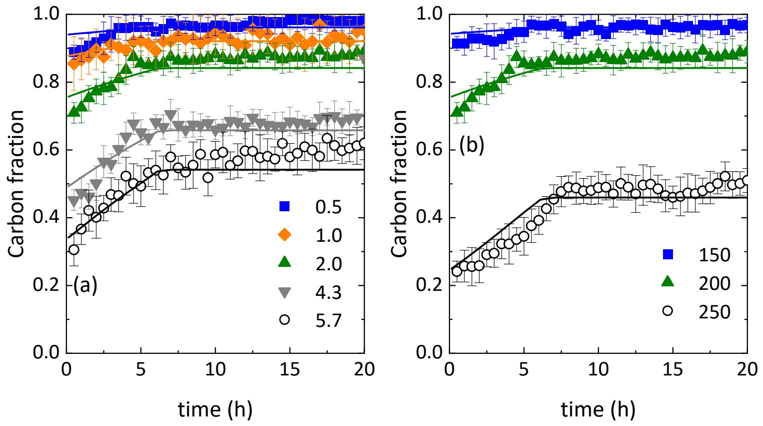

Figure S6. Comparison of experimental and predicted evolution with time of 1-butene carbon fraction at the outlet of the reactor with the VLE-based models and deactivation equation “a” (a) at 200 ºC using different space time values (in g h mol_C^{-1}) and (b) at different temperatures (in ºC) using a space time value of 2.0 g h mol_C^{-1}. Total pressure of 40 bar and 1-butene partial pressure of 28 bar in all cases.

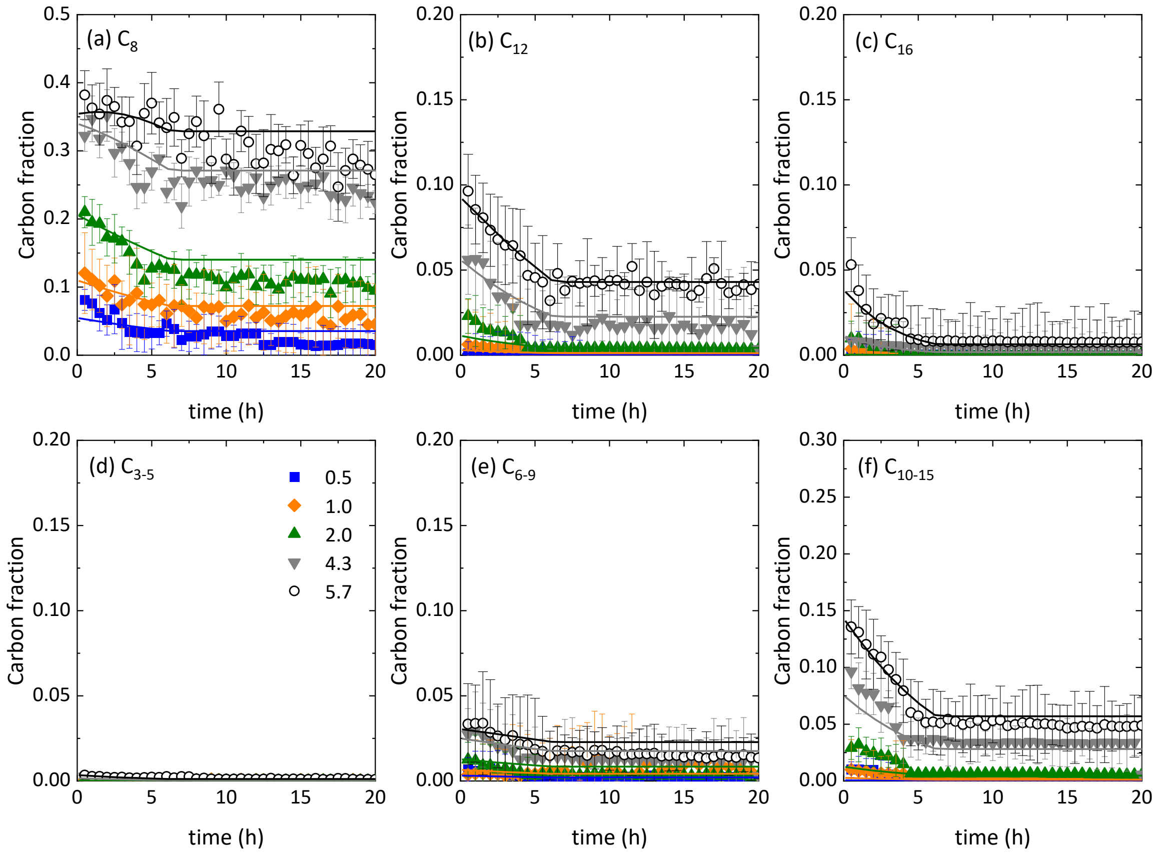

Figure S7. Comparison of experimental and predicted evolution with time of (a) C_8, (b) C_{12}, (c) C_{16}, (d) C_{3-5}, (e) C_{6-9}, (f) C_{10-15} olefin carbon fractions at the outlet of the reactor with the VLE-based model and deactivation equation "a" at 200 ºC using different space time values (in g h mol_C^{-1}). Total pressure of 40 bar and 1-butene partial pressure of 28 bar in all cases.

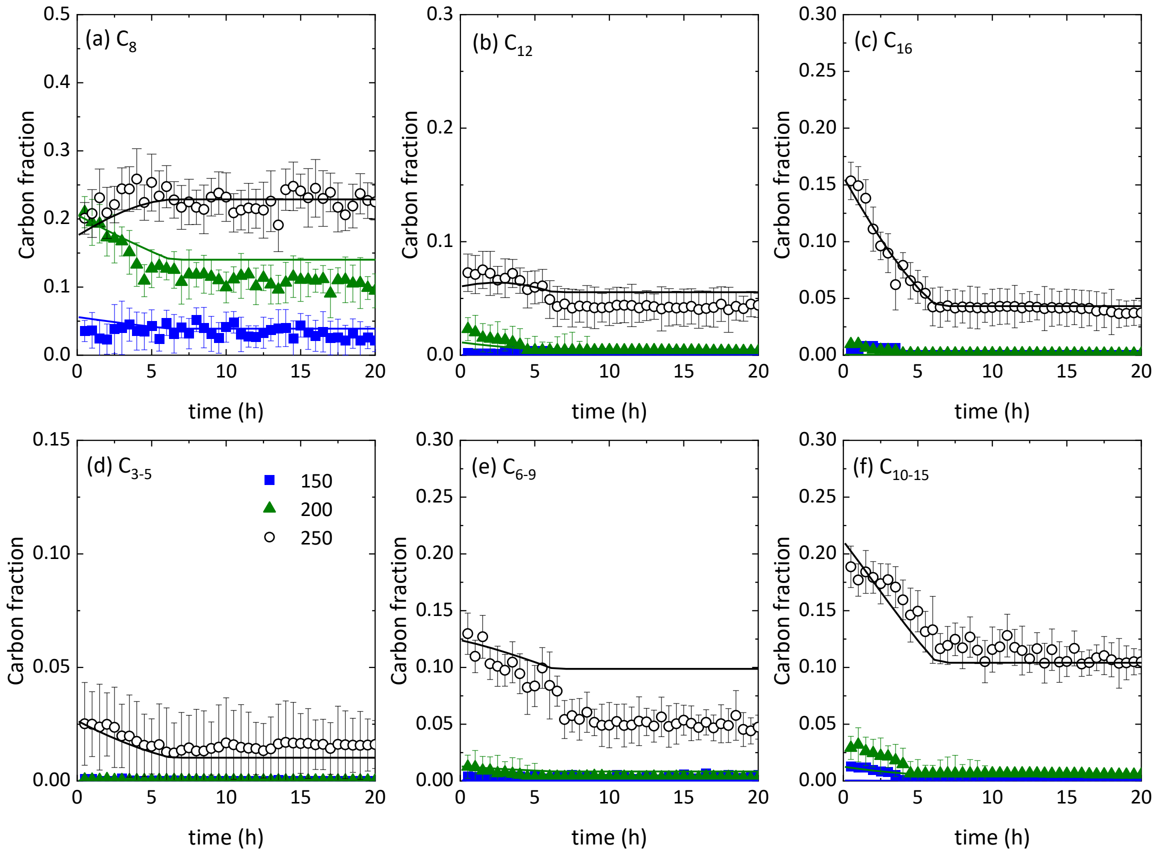

Figure S8. Comparison of experimental and predicted evolution with time of (a) C_8, (b) C_{12}, (c) C_{16}, (d) C_{3-5}, (e) C_{6-9}, (f) C_{10-15} olefin carbon fractions at the outlet of the reactor with the VLE-based model and deactivation equation "a" at different temperatures (in ºC) using a space time value of 2.0 g h mol_C^{-1}. Total pressure of 40 bar and 1-butene partial pressure of 28 bar in all cases.

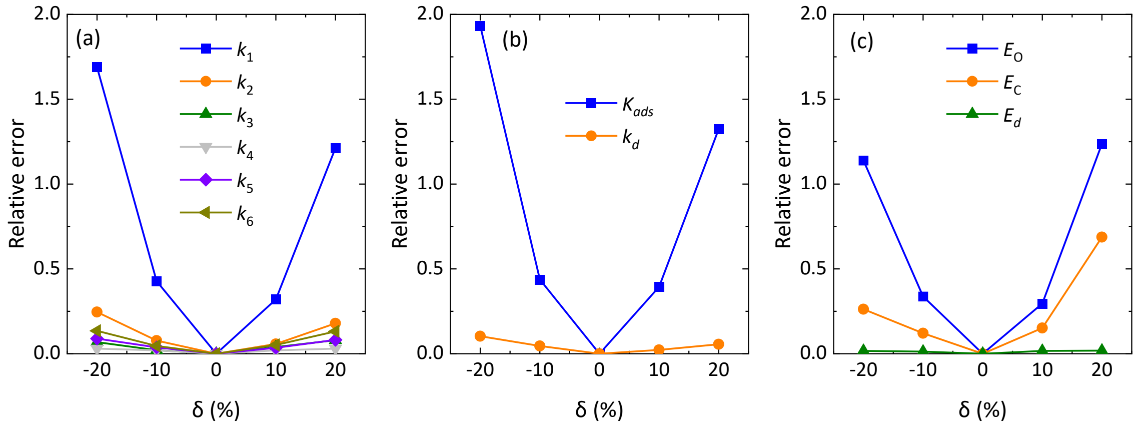

Figure S9. Sensitivity analysis of the kinetic parameters: (a) kinetic constants, (b) adsorption equilibrium and deactivation constants and (c) apparent activation energies. Relative statistical error for ±20% perturbation of each parameter optimized value.

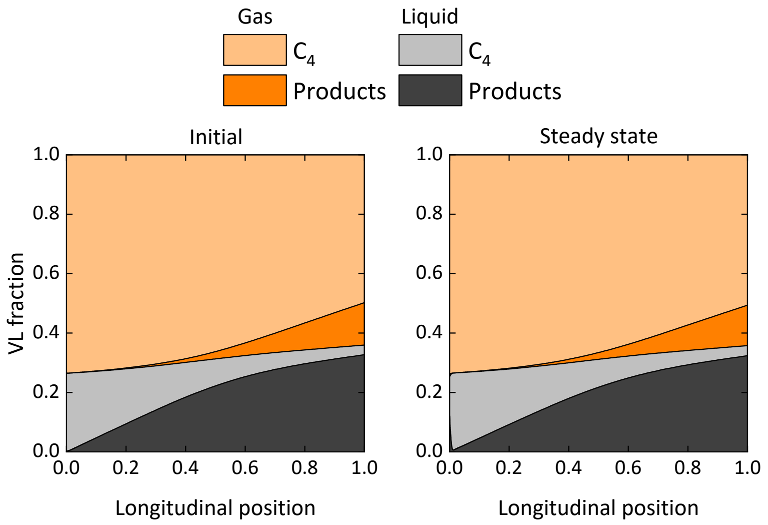

Figure S10. Simulations of the vapor and liquid fraction evolution with the reactor bed length at 250 ºC using a space time value of 100 g h mol_C^{-1}. Total pressure of 1 bar and 1-butene partial pressure of 0.8 bar in all cases.

Table S2. Kinetic parameters for the gas-phase model with deactivation equation “a”.

	**Value**
k_1^* (mol_C g^{-1} h^{-1} bar^{-2})	$1.6\ 10^{-3} \pm 7.3\ 10^{-5}$
k_2^* (mol_C g^{-1} h^{-1} bar^{-2})	$2.5\ 10^{-3} \pm 3.6\ 10^{-4}$
k_3^* (mol_C g^{-1} h^{-1} bar^{-2})	$1.4\ 10^{-2} \pm 8.0\ 10^{-3}$
k_4^* (mol_C g^{-1} h^{-1} bar^{-1})	$8.3\ 10^{-4} \pm 4.5\ 10^{-4}$
k_5^* (mol_C g^{-1} h^{-1} bar^{-2})	$4.7\ 10^{-5} \pm 1.5\ 10^{-5}$
k_6^* (mol_C g^{-1} h^{-1} bar^{-2})	$2.9\ 10^{-3} \pm 4.3\ 10^{-5}$
E_O (kJ mol^{-1})	$4.3\ 10^{1} \pm 4.3\ 10^{0}$
E_C (kJ mol^{-1})	$1.3\ 10^{2} \pm 9.5\ 10^{0}$
K_{ads} (bar^{-1})	$1.8\ 10^{0} \pm 4.0\ 10^{-2}$
k_d^* (h^{-1} bar^{-1})	$8.9\ 10^{-2} \pm 1.2\ 10^{-3}$
E_d (kJ mol^{-1})	$1.0\ 10^{-1}$

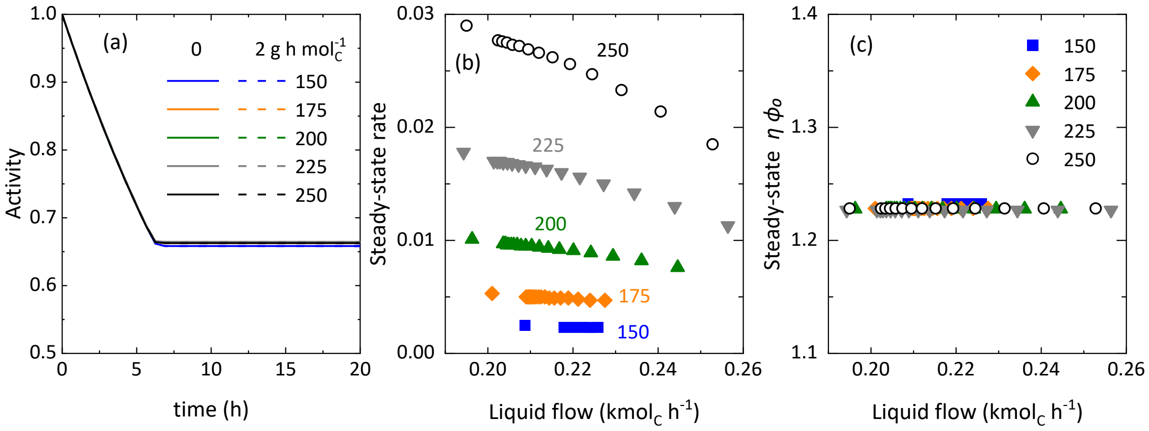

Fig S11. (a) Evolution with time of the activity at different temperatures (in ºC) and space times values of 0 and 2 g h mol$_C^{-1}$. Effect of the flow of liquid products in the reactor on the steady-state (b) 1-butene self-oligomerization rate (r_1) and (c) $\eta\phi_0$ values at different temperatures (in ºC) using the VLE-based model and deactivation equation "i". Total pressure of 40 bar and 1-butene partial pressure of 28 bar in all cases.

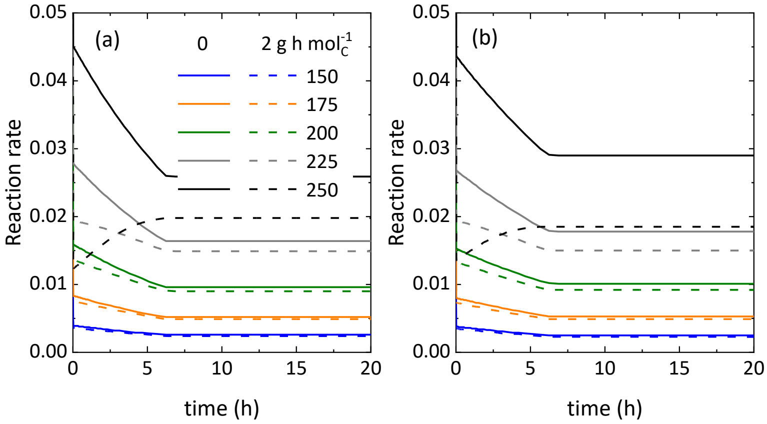

Figure S12. Evolution with time of 1-butene self-oligomerization reaction (r_1) at different temperatures (in ºC) and space time values of 0 and 2 g h mol_C^{-1} using the VLE-based models and deactivation equations (a) "a" and (b) "i". Total pressure of 40 bar and 1-butene partial pressure of 28 bar in all cases.

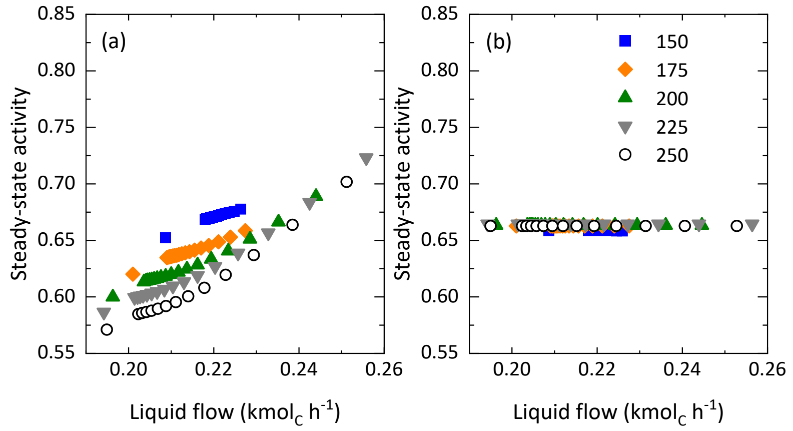

Figure S13. Effect of the flow of liquid products in the reactor on the steady-state activity values at the outlet of the reactor at different temperatures (in ºC) using the VLE-based model and deactivation equations (a) "a" and (b) "i". Total pressure of 40 bar and 1-butene partial pressure of 28 bar in all cases.

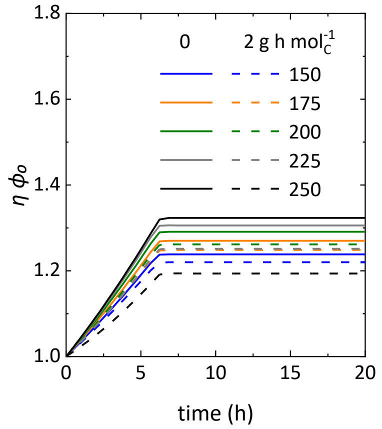

Figure S14. Evolution with time of $\eta\phi_0$ values at different temperatures (in ºC) and space time values of 0 and 2 g h mol$_C^{-1}$ using the VLE-based model and deactivation equation "a". Total pressure of 40 bar and 1-butene partial pressure of 28 bar in all cases.
